# Experiments on Adversarial Examples for Deep Learning Model Using Multimodal Sensors

**DOI:** 10.3390/s22228642

**Published:** 2022-11-09

**Authors:** Ade Kurniawan, Yuichi Ohsita, Masayuki Murata

**Affiliations:** 1 Graduate School of Information Science and Technology, Osaka University, Suita 565-0871, Osaka, Japan; 2 Institute for Open and Transdisciplinary Research Initiatives, Osaka University, Suita 565-0871, Osaka, Japan

**Keywords:** adversarial examples, generative adversarial networks, multimodal sensors

## Abstract

Recently, artificial intelligence (AI) based on IoT sensors has been widely used, which has increased the risk of attacks targeting AI. Adversarial examples are among the most serious types of attacks in which the attacker designs inputs that can cause the machine learning system to generate incorrect outputs. Considering the architecture using multiple sensor devices, hacking even a few sensors can create a significant risk; an attacker can attack the machine learning model through the hacked sensors. Some studies demonstrated the possibility of adversarial examples on the deep neural network (DNN) model based on IoT sensors, but it was assumed that an attacker must access all features. The impact of hacking only a few sensors has not been discussed thus far. Therefore, in this study, we discuss the possibility of attacks on DNN models by hacking only a small number of sensors. In this scenario, the attacker first hacks few sensors in the system, obtains the values of the hacked sensors, and changes them to manipulate the system, but the attacker cannot obtain and change the values of the other sensors. We perform experiments using the human activity recognition model with three sensor devices attached to the chest, wrist, and ankle of a user, and demonstrate that attacks are possible by hacking a small number of sensors.

## 1. Introduction

Artificial intelligence (AI) based on deep neural networks (DNNs) has significantly impacted human lives by making them more secure, efficient, automated, and accurate. Currently, AI is widely used in many areas, such as smart nations [1], agriculture [2], medicine [3], industry [4], and human activity recognition (HAR) [5]. Many Internet of Things (IoT) sensor devices are used to achieve accurate recognition of the real world. Observations from these devices are collected via the Internet or a network managed by the service provider. The machine learning model then recognizes the current situation using the collected observations as its input. For example, an autonomous vehicle recognizes the surroundings using multimodal sensors, such as cameras, radar, LiDAR, global navigation satellite system (GNSS), gyroscopes, and magnetometers [6]. Many sensors have been used in HAR. Ichino et al. used accelerometers, gyroscopes, magnetometers, and electrocardiogram sensors to recognize human activities [7]. Debauche et al. also proposed a model to recognize human activities based on accelerometer and gyroscope signals [8].

Although we celebrate advances in AI and sensors, state-of-the-art DNNs are vulnerable to adversarial examples [9]. Adversarial examples are inputs designed by an adversary that cause a machine learning system to generate incorrect outputs [10]. By creating an incorrect output, an attacker can degrade the service that is based on AI. If the service is related to users’ health, the degradation of the service may have a significant impact on the users’ health. By attacking such services, an attacker may consider users as hostages. Some studies have demonstrated that an attacker can fool machine learning models for HAR, which is closely related to healthcare [11,12,13].

Considering an architecture using multiple sensor devices, hacking a small number of sensors creates a significant risk. As the number of sensor devices in a system increases, the risk of attack by hacking some of these sensors increases. Some sensors may be located near people. As a result, attackers may easily access them, find vulnerabilities, or replace them. In fact, an attack on wearable sensors has already been demonstrated [14].

The attacker may attack the machine learning model using the hacked sensor. Even if an attacker can hack only a small part of the sensor, the sensor may have a large impact on the machine learning model. However, the impact of hacking a small part of the sensor has not been discussed thus far. Some papers demonstrated that adversarial examples on the DNN model based on IoT sensors are possible, but with the assumption that an attacker can access all features of the model.

Therefore, we discuss the possibility of attacks on DNN models by hacking a small number of sensors through experiments. In this experiment, we assume that the attacker first hacks the sensor device. The attacker can obtain the values of the hacked sensors and change them but cannot obtain and change the values of the other sensors. In this study, we demonstrate that an attacker can manipulate a DNN model, even in this case.

To demonstrate the attacks, we introduce a generator that generates adversarial examples when a small number of sensor devices are hacked. The generator uses the values from the hacked sensors as inputs and generates perturbations so that the features, including the perturbations, are classified into the target class by the target model.

In our experiments, we use an open dataset for HAR based on three sensor devices attached to the chest, wrist, and ankle of the subjects and demonstrate that the attacker can change the output of the target model by hacking only one of those devices.

In summary, our main contributions are as follows:

- We formulate the adversarial example by monitoring and changing the values of a part of the sensors.
- We demonstrate that adversarial examples are possible even if the attacker can monitor and change only a part of the sensors.

The rest of this paper is organized as follows. We discuss the work related to our research in Section 2. In Section 3, we describe the attack definition and how to generate the attacks. Section 4 presents adversarial attack examples for deep learning models using multimodal sensors. The results are discussed in Section 5. Finally, we conclude the paper in Section 6.

## 2. Related Work

Szegedy et al. coined the phrase “adversarial example,” and since then the number of publications related to adversarial examples has increased exponentially.

Several methods for generating adversarial examples have been proposed, as shown in Table 1, such as the fast gradient sign method (FGSM) [9], basic iterative method (BIM) [15], saliency map method [16], FGSM [9], and Carlini–Wagner method (C&W) [17] and AdvGAN [18]. FGSM and BIM are examples of white-box attacks that access an entire target model. Gradient-based attack methods, such as FGSM, determine the maximum constrained max-norm perturbation of $x^{\prime }=x+\varepsilon .sign\nabla_{x}\ell \left(x,y\right)$ by computing the gradient of the input’s loss function $\nabla_{x}\ell \left(x,y\right)$ by multiplying a small chosen constant ε by the gradient’s sign vector. Carlini and Wagner attacks [17] and other optimization-based methods optimize adversarial perturbations, subject to several constraints. This method focuses on the L_0_, L_2_, and L_∞_ distance metrics and generates perturbations by minimizing the loss function under the constraint that the distance metrics of the perturbations are less than a predefined threshold. Although optimization-based methods generate adversarial perturbations that fool the target model without violating the constraints, they take a long time because the optimization problem must be solved to generate each perturbation.

Another approach for generating adversarial examples is to train the generator. Xiao et al. [18] proposed adversarial examples using GAN architecture to efficiently generate more realistic adversarial examples. This was followed by [19,20,21]. They proposed training a feed-forward network that generates perturbations to create diverse adversarial examples and a discriminator network to ensure that the generated examples are realistic. Once the generator is trained, adversarial perturbations can be efficiently generated.

In this study, we used a method based on the generator. However, we assume that an attacker can obtain only the values of the hacked sensors, whereas existing studies assume that all features can be used as the input of the generator, as shown in Table 1.

Potential adversarial examples have also been discussed in many critical applications. Table 2 shows the papers demonstrating adversarial examples. Finlayson et al. demonstrated the danger of adversarial attacks in the medical domain [22]. By taking input from the vision sensor and adding adversarial noise to a dermatoscopy image, they successfully changed the patient’s diagnosis from benign to malignant or vice versa. Han et al. demonstrated an attack on a deep learning model based on a raw signal electrocardiogram (ECG) [23]. Benegui et al. successfully attacked a DNN model for user identification based on motion sensors and converted a discrete three-axis raw signal sensor into a grayscale image representation [12]. Sah et al. demonstrated attacks on a machine learning model for HAR based on multiple wearable sensors [13]. They generated adversarial examples at the raw-signal level and discussed their transferability. In this study, we use the same dataset as Sah et al. to demonstrate the possibility of an attack, but the attack scenario is different; we assume that the attacker can only access the hacked sensors, whereas Sah et al. assumed that the attacker could access all raw signals directly.

## 3. Definition of Adversarial Examples by Hacking a Small Number of Sensors

### 3.1. Definition of Attack

**Figure 1:**
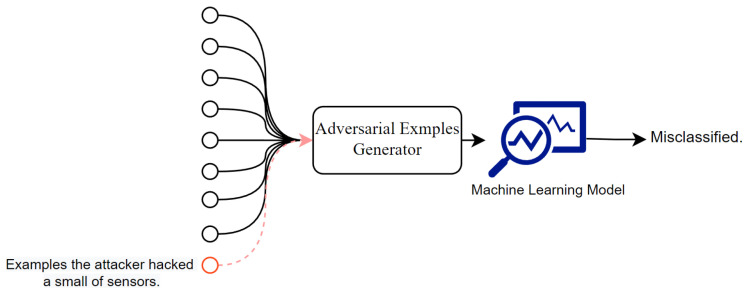
Overview of the attacks.

We focus on a system that gathers values from multiple sensors and performs classification tasks based on a machine learning model. An attacker against this system hacks some sensors; the attacker may hack sensors with the same vulnerabilities but cannot hack other sensors. An attacker can obtain the values of the hacked sensors and change them. The objective of the attack is to cause misclassification by changing the values of the hacked sensors, as shown in Figure 1.

Hereafter, we define $f\left(x_{0:t}\right)$ as a function of the target model; $x_{0:t}=\left(x_{0},x_{1},\ldots ,x_{t}\right)$ is the input of the target model constructed from the sensor values obtained from time 0 to time $t^{\prime };$ and $x_{t}$ is the vector corresponding to the sensor values at time t. $f\left(x_{0:t}\right)$ indicates the classification result at time t; we denote the jth element of the output of the model by $f_{j}\left(x_{0:t}\right),$ and $f_{j}\left(x_{0:t}\right)$ indicates the probability that the situation at time t is classified into the ith class.

The attacker can monitor and change the features from the hacked sensors. We define the vector $B=\left(b_{1},b_{2},\ldots ,b_{m}\right)$ indicating the features from the hacked sensors; $b_{i}=1$ if the ith feature is from the hacked sensor. Using B, the features monitored by the attacker at time t are ${\dot{x}}_{t}=B\circ x_{t}$, where ∘ indicates the element-wise product.

The attacker generates the perturbations so that the classification results become the target class. That is, the objective of the attacker is ${\mathrm{argmax}}_{i}\;f_{i}\left({x^{\prime }}_{0:t}\right)=C,$ where ${x^{\prime }}_{0:t}=\left({x^{\prime }}_{0,},{x^{\prime }}_{1},\ldots ,{x^{\prime }}_{t}\right)$ is the input feature after adding the perturbation at time t and C is the target class.

The attacker generates perturbations based only on the features from the hacked sensors. That is, the attacker uses ${\dot{x}}_{t}$. We denote ${\dot{x}}_{0:t}=\left({\dot{x}}_{0},{\dot{x}}_{1},\ldots ,{\dot{x}}_{t}\right)$. We define a function $G\left({\dot{x}}_{0:t}\right)$ whose inputs are the features monitored by the attacker and whose outputs are the generated perturbations. By adding perturbations generated by the generator $G\left({\dot{x}}_{0:t}\right)$, the features that include the attacks become $x_{t}^{\prime }=x_{t}+B\times G\left({\dot{x}}_{0:t}\right)$

In this study, we assume that the attacker has enough information about the target model and some knowledge of the sensors. The attacker can obtain the same model if the target uses an open model. Even if the model is not open, the information on the model can be extracted by conducting a model extraction attack [24], which steals the architecture, parameters, and hyperparameters of the target by monitoring the model’s output if the attacker can use the model. This paper assumes the case after obtaining accurate information on the target model. However, the stolen model may include estimation errors. The demonstration of the attacks in the case that the information of the target model is inaccurate is one of our future works.

On the other hand, knowledge of the sensors can be obtained through generally known knowledge. If it is generally known that values of a sensor correlate with the other sensors’ values, attackers can use this knowledge. If the attackers can buy and use the same type of sensors, they can perform experiments to obtain the knowledge of the sensors. In this paper, we model the knowledge of the sensors by an estimator ${\hat{x}}_{t}=S\left({\dot{x}}_{t:0}\right)$ whose input is the feature that can be monitored by the attacker and whose output is all features.

### 3.2. Generation of Attack

**Figure 2:**
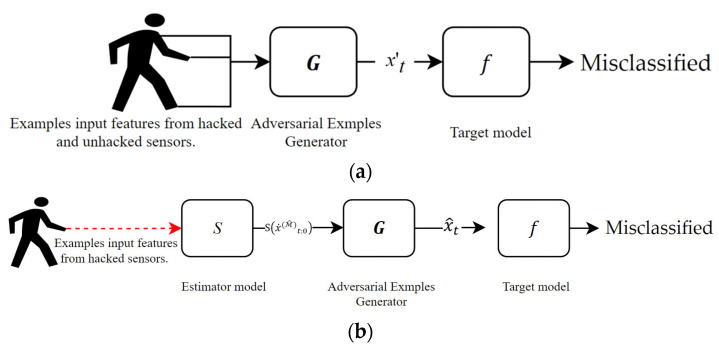
Overview of architecture generated an adversarial example in multimodal sensors. Figure (**a**) shows generated an adversarial attack using a generator model, and figure (**b**) process trained the generator.

In this study, the attack is generated using the generator $G\left({\dot{x}}_{0:t}\right),$ as shown in Figure 2a.

The generator $G\left({\dot{x}}_{0:t}\right)$ is trained in advance. The attacker can monitor the features of the target from the hacked sensors. We denote the dataset monitored from the hacked sensors as M. The attacker also has some knowledge of the other sensors and can estimate the values of the other sensors using the estimator $S\left({\dot{x}}_{t:0}\right)$, although the estimation may be inaccurate. Using the dataset M and estimator $S\left({\dot{x}}_{t:0}\right)$, the attacker can generate the dataset that can be used to train the generator. Hereafter, we denote the generated training data as $\hat{M}$. Each element of $\hat{M}$ can be generated by:

(1) $${\hat{x}}_{t}=S\left({\dot{x}}^{\left(M\right)}{}_{t:0}\right),$$

where ${\dot{x}}^{\left(M\right)}{}_{0:t}=\left({\dot{x}}^{\left(M\right)}{}_{0},{\dot{x}}^{\left(M\right)}{}_{1},\ldots ,{\dot{x}}^{\left(M\right)}{}_{t}\right)$, and ${\dot{x}}^{\left(M\right)}{}_{t}$ is an element of data in the dataset M.

Figure 2b shows the process to train the generator using the dataset $\hat{M}$. When training the generator, the attacker has the information on the target model $f\left(\right)$. Using $f\left(\right)$, the attacker trains the generator by minimizing the following loss function:

(2) $$\ell_{adv}^{f}=E_{x_{t}}\;\left[\ell_{f}\left(f\left({\hat{x}}_{t}+B\circ G\left({\hat{x}}_{0:t}\right)\right),C_{t}\right)\right],$$

where ${\hat{x}}_{0:t}=\left({\hat{x}}_{0},{\hat{x}}_{1},\ldots ,{\hat{x}}_{t}\right)$, $\ell_{f}\left(y,C\right)$ is the loss function of the target model when the output of the target model is y, the target class is C, and $C_{t}$ is the target class at time t. By generating the perturbation using the generator trained to minimize this loss function, the features after the attack can be classified into the attacker’s desired class.

## 4. Experiments

### 4.1. Target Scenario

#### 4.1.1. Overview

In this scenario, we use as a target model a machine learning model that identifies human activities from three sensors. This model is used to recognize human activities for healthcare, smart-home environment, and so on. In this model, the user wears three sensor devices on the chest, left ankle, and right wrist. All three sensor devices have 3D accelerometers. Moreover, the sensor device on the chest has an ECG sensor, and the other sensor devices have 3D gyroscopes and 3D magnetometers. The sensor devices send their monitored values to the server with the machine learning model based on a DNN. The server recognizes the user’s current activity by handling time-series data sent from the sensors.

In this experience, we focus on specific subjects as the target and generate perturbations so that the activities of the specific subjects are identified as the target classes, which are different from the ground-truth classes.

To generate the perturbations, we assume that the attacker has hacked one of the sensor devices, the ankle sensor. The attacker can access and change the sensor values of the ankle sensor but cannot access the values of the other sensors. By changing the sensor values sent to the server, the attacker attempts to change the activity recognized by the machine learning model.

In this study, we assume that the attackers use their knowledge to train the attack generator. In this scenario, we simulate the attacker’s knowledge using an estimator trained by a dataset without the target subjects. By changing the amount of dataset used to train the estimator, we simulate the various cases—from the case that the attacker has accurate knowledge to the case in which the attacker has inaccurate knowledge.

#### 4.1.2. Dataset

We used an open dataset called the MHealth dataset [25]. This dataset includes 12 physical activities (standing, sitting, lying down, walking, climbing stairs, bending forward, lifting arms forward, knees, cycling, jogging, running, and jumping back and forth) for ten subjects. They used wearable sensor devices located on the subject’s chest, right wrist, and left ankle, and recorded the sensor values with a sampling frequency of 50 Hz. Table 3 lists the sensors used in the dataset.

The Mhealth dataset includes the time-series of sensor values. From this dataset, we extract the data with a length of 500 used for training and validation by using a sliding window. The number of extracted data for each subject and each class is shown in Table 4.

Among ten subjects, we used subjects 9 and 10 as the target subjects. The data from the other subjects were used to train the target model and the estimator, but the data from the target subjects were not used to train the target model and estimator. When training the generator, we used the data of the target subjects but only the features of the hacked sensors, assuming that the attacker can access the values of the hacked sensors of the target subjects. The values of the other sensors used to train the generator are obtained using the estimator.

#### 4.1.3. Target Model

**Figure 3:**
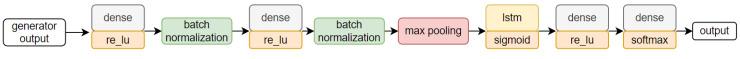
The architecture of the target model.

This paper uses a model based on the long short-term memory network (LSTM) architecture proposed for HAR [26]. Figure 3 shows the architecture of the target model used in this study.

We built the target model on top of TensorFlow and Keras and trained it using multiple NVIDIA Quadro RTX 5000. We trained the model to minimize the cross-entropy of the outputs and the corresponding labels in the training data using the Adam optimizer with a learning rate of 0.001, batch size of 32, and 100 epochs. Data from eight subjects were used to train the model. The data from the remaining subjects were used to evaluate the target model and the attack. To train the target model, we used time-series data generated by dividing the time-series data included in the training data into small sets of time-series data with lengths of 500 using the sliding-window technique.

#### 4.1.4. Estimator

**Figure 4:**
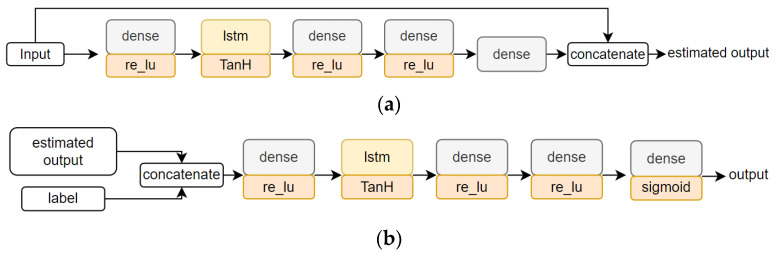
Estimation model architecture: estimator model on (**a**) and discriminator model on (**b**).

In this study, we constructed an estimator to simulate the attacker’s knowledge. Estimator S estimates sensor values that are not obtained by the attacker from the values of the hacked sensors. In this study, we used the conditional-GAN training technique [27] to train estimator S, as shown in Figure 4.

In this technique, the discriminator D  is introduced. The discriminator D distinguishes the output of the estimator from the training dataset. By training S to generate values that cannot be distinguished by discriminator D, we construct S to estimate the original values.

The discriminator D and the estimator S  are trained alternatively. When training discriminator D, the parameters of discriminator D are updated to minimize the loss function, indicating the accuracy of the classification using the original values and the values generated by S as the training dataset. However, when training estimator S, we input the features of the hacked sensors of the training data to S, obtain the output from S indicating the estimated features, including the values of the other sensors, and use the output from S as the input for D. Finally, the output from D is obtained. Based on the output from D obtained by this process, the parameters of S are updated to minimize the same loss function of D by setting the target class of the generated values to the class for the original data.

We use LSTM in the estimator and discriminator to handle the time-series data. Figure 4 also shows the structures of the estimator and discriminator used in this demonstration. In the estimator, the values of the other sensors were estimated using the CNN–LSTM structure. Then, at the final layer, the estimator estimates the features of all the sensors, including the values of the hacked sensors, by concatenating the estimated sensor values and the values that can be monitored.

In this study, TensorFlow and Keras with multiple NVIDIA Quadro RTX 5000 were used. We trained the estimator and discriminator using 50 iterations with a learning rate of 0.002 and a batch size of 128. To train them, binary cross-entropy was used as the loss function. The data used to train the estimator and discriminator were generated by dividing the long time-series data using the sliding-window technique.

#### 4.1.5. Generator

**Figure 5:**
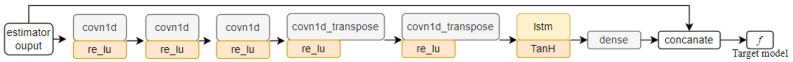
The architecture of the generator model.

In this section, we use the generator based on 1D CNN and LSTM to handle the time-series data of the hacked sensors as inputs. Figure 5 shows the generator used in this study.

We trained the generator using features estimated by the estimator from the values of the hacked sensors of the target subjects. We used Adam optimization and set the batch size to 256, learning rate to 0.002, epochs to 1, and epsilon ε to 0.3. Similar to the target model and estimator, we divided the time-series of features into time-series features with a sliding-window size of 500 and used the divided time-series features to train the generator.

### 4.2. Property of the Target Model

Before demonstrating the attacks, we investigated the properties of the target model by comparing it with a similar model using only one sensor device. In this comparison, we used precision and recall as metrics. Precision and recall are defined as

(3) $$Precision=\frac{tp}{tp+fp}\;$$

(4) $$Recall=\frac{tp}{tp+fn}$$

where true positive (tp) is the number of data that can be classified correctly, false positive (fp) is the number of data that are classified into a class but whose correct class is different. False negative (fn) is the number of data not classified into a class but whose correct class is the class.

Table 5 lists the precision and recall values for each class. This table shows that the model using only a single sensor device cannot recognize some classes. For example, the chest sensor cannot distinguish between standing and sitting states. The recall result shows that the wrist sensor cannot accurately recognize the walking class. The ankle sensor has high precision and recall compared with other sensors. However, even the ankle sensors achieve only 88% recall in the standing class. In contrast, the target model using all three sensors can recognize any class. In other words, multiple sensor devices are required for HAR.

We also investigate the impact of each sensor on the classification results. We use an integrated gradient [28]. The integrated gradient is a method for evaluating the impact of each feature on the results of a machine learning model.

The integrated gradient of the ith feature of the input x on the class j is defined as

(5) $${\mathrm{IntegratedGrads}}_{ij}(x)::=\left(x^{\left(i\right)}-{x^{\prime }}^{\left(i\right)}\right)\times \int_{\alpha =0}^{1}\frac{\partial f_{j}\left(x+\alpha \times \left(x-x^{\prime }\right)\right)}{\partial x^{\left(i\right)}}\;d\alpha$$

where $x^{\prime }$ represents the baseline input, $x^{\left(i\right)}$ is the ith feature of x, and α  is the interpolation constant. The features whose integrated gradient is far from zero have a significant impact on the output of the model. We calculate the integrated gradient for data of the target subject and calculate the average of them for each class.

**Figure 6:**
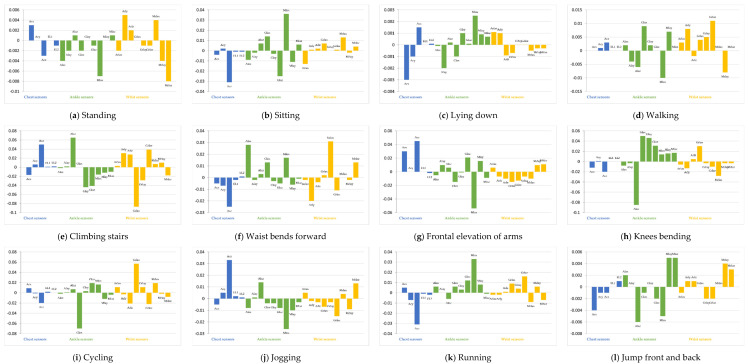
Impact result of input feature sensors for each class in the target model using integrated gradients (IG).

Figure 6 shows the integrated gradient for 12 classes. The vertical axis in Figure 6 is the integrated gradient for each feature. A large positive integrated gradient means that the feature has a strong positive correlation to the class, and a large negative integrated gradient means that the feature has a strong negative correlation. If the integrated gradient is close to 0, the corresponding feature does not contribute to the classification.

Figure 6a indicates that Mlax from the ankle sensor and Arly, Mrlax, and Mrlaz from the wrist sensor have a strong correlation to the classification into Standing class and have a large impact on the classification results. Similarly, the other figures in Figure 6 indicate the features that contribute to the classification. From this figure, multiple sensors contribute to classification into any classes in the target model. Namely, our target model identifies all classes based not only on specific sensor devices, but also on multiple sensor devices.

### 4.3. Property of the Estimator

In Table 6, we show the results of the estimator trained using five and three subjects. In this table, we evaluate the accuracy of the estimator using the mean square error (MSE) as a metric. The MSE is defined as

(6) $$\mathrm{MSE}=\frac{\sum {}_{t=1}^{n}\sum {}_{i\in \left\{i|o_{i}=1\right\}}\left(x_{t,i}\right.-{\left.{\hat{x}}_{t,i}\right)}^{2}}{n},$$

where $x_{t,i}$ and ${\hat{x}}_{t,i}$ are the actual and estimated values of the ith feature at time t, and n is the length of the validation data. The smaller the MSE value is, the more accurate the prediction results are.

In this study, we trained the estimator using three and five subjects and then evaluated the accuracy of the data using data from two subjects that were not included in the training dataset for the target model and the estimator. Table 6 shows that the evaluation errors increased as the number of subjects used to train the estimator decreased. In the remainder of this section, we investigate whether attackers with these estimators can succeed in the attack.

### 4.4. Demonstration of the Attack

**Figure 7:**
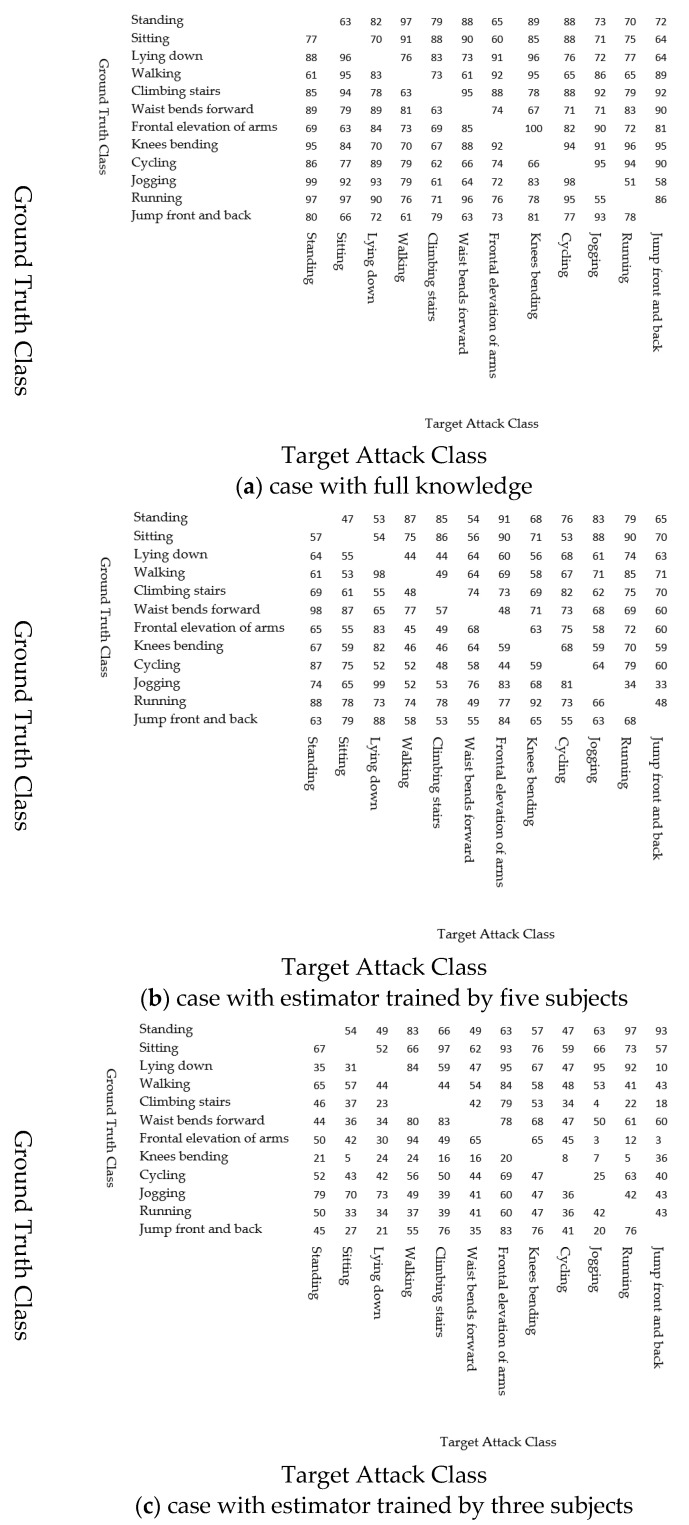
(**a**) Results of attacks on ankle sensors using full knowledge; (**b**) results of success attack rate using five subjects; and (**c**) results of success attack rate on ankle group sensor using three subjects.

In this section, we describe the attacks. To evaluate the generated attacks, we introduce a metric called the attack success ratio, which is defined as

$$\frac{N^{success}}{N^{attack}}\;,$$

where $N^{attack}$ is the total number of time slots, including the attacks, and $N^{success}$ is the number of time slots in which the results of the target model are the attacker’s desired classes. Figure 7 shows the results. The rest of this subsection discusses the results.

#### 4.4.1. Case That the Attacker Has Full Knowledge of All Sensors

Before discussing the results of the impact of the attacker’s knowledge, we first investigate the case in which the attacker has sufficient knowledge. In this case, we use the actual values of all the sensors to train the generator instead of using the estimated values. Note that even in this case, the attacker does not have the values of the other sensors during attacks but can monitor only the features from the hacked sensors.

Figure 7a shows the results that the attack success ratio depends on the ground truth and target classes. For example, all attacks from frontal elevation of arms to knee bending succeeded, whereas the attack success ratio of attacks from jogging to running was low. However, even in the case with the lowest success ratio, more than half of the attacks succeeded. That is, the attacks succeeded by changing the values of the ankle sensors, although the other sensors also have a large impact on the classification results.

We discuss the property of the generated attacks in Section 4.5.

#### 4.4.2. The Impact on the Attacker’s Knowledge

Figure 7b,c show the attack success ratio for the cases in which the attacker has the estimator trained by five and three subjects, respectively. As discussed above, as the number of subjects used to train the estimator decreases, the estimation errors increase. Consequently, the attack success ratio also decreases.

Figure 7c also indicates that the attacks for some classes succeeded even if the attacker did not have accurate information on the other sensors. For example, the attacks from frontal elevation of arms to walking succeeded with a high attack success ratio, while most attacks from frontal elevation of arms to jogging or jumping front and back failed. We discuss the cause of these differences in the results in Section 4.5.

### 4.5. Property of the Generated Attacks

**Figure 8:**
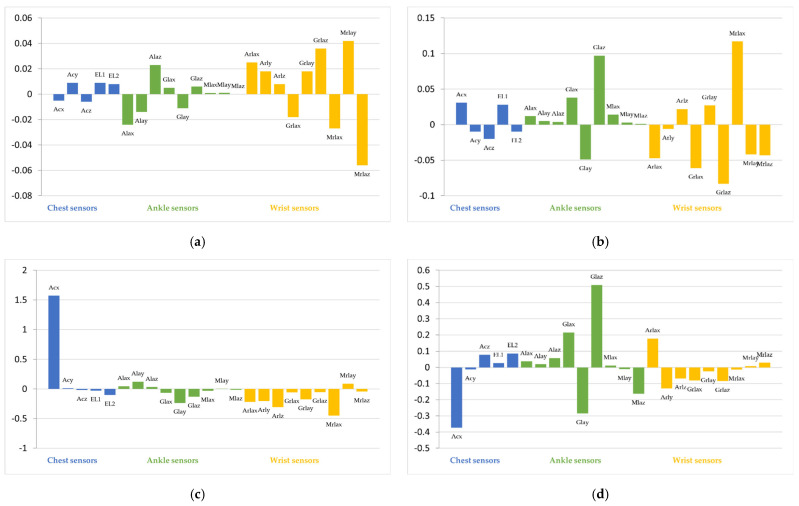
Integrated gradients of the inputs that include attacks. (**a**) Ground Truth: Frontal elevation of arms, Target: Walking (case with full knowledge). (**b**) Ground Truth: Frontal elevation of arms, Target: Jogging (case with full knowledge). (**c**) Ground Truth: Frontal elevation of arms, Target: Jump front and back (case with full knowledge). (**d**) Ground Truth: Frontal elevation of arms, Target: Walking (case with estimator trained by three subjects).

In this section, we discuss the properties of the generated attacks. We calculate the IGs of the generated attacks. Figure 8 shows examples of the average integrated gradients of the attack generated for data whose ground-truth class is frontal elevation of arms.

In Figure 8, compared with Figure 6, the features that include attacks that have a large impact on the classification are very different from the data without attacks. This is because the generator does not generate the perturbation to make the input of the model similar to the normal data whose class is the target class. However, the generator generates the perturbations to minimize the loss function of the target model. As a result, the features are very different from the original data but are classified into the target class by the target model.

Figure 8 also shows that the features of the sensors that are not hacked have a large impact on the classification results in some cases. In this case, by changing the features from the hacked sensor, the attacker moves the features to a location corresponding to the target class in the feature space. Among the changed features, the features from the other sensors contribute to the classification of the target class. However, to succeed in this type of attack, the attacker must know the impact of the features from the other sensors on the classification results. That is, accurate information from other sensors is required.

However, as shown in Figure 7, even if the attacker’s knowledge is inaccurate, attacks on certain classes succeed. One example is the attack from frontal elevation of arms to walking. Figure 8d shows the average IGs of the data with attacks generated in the case with the estimator trained with three subjects. Figure 8d indicates that the features from the ankle sensor have the largest impact on the classification results; therefore, this attack succeeded even if the attacker does not have accurate knowledge of the other sensors. In this case, the attacks cause the classification into the target class by increasing the contributions of the features of the hacked sensors for the target class. The attacker can determine and change the values of the features of the hacked sensors. That is, the attacker can accurately calculate the contributions of the features of the hacked sensors and change the features to increase the contributions. As a result, this attack succeeds even if the attacker does not have sufficient information from other sensors.

## 5. Discussion

In this study, we demonstrate that attacks that cause misclassification in target models are possible even if the attacker hacked a part of the sensors. In particular, if the attacker has sufficient knowledge of the other sensors, the attack succeeds with a high probability, although the attacker cannot monitor the current values of the other sensors. We also demonstrate that the attacks succeed in some cases, even if the attacker does not have sufficient knowledge of other sensors.

In our experiment, we focus on one model as a target model. However, our approach is not based on any assumptions about the target model. Thus, this kind of attack is possible in the other models, though demonstration using the other models and a different dataset is one of our future research topics.

In this study, we assume that the attacker has some knowledge of the legitimate sensors and we simulated this knowledge by using the estimator. By using the estimator trained by a limited amount of dataset, we simulated the case that the attacker’s knowledge is inaccurate. In the actual situation, the attackers may obtain knowledge of the legitimate sensors by using generally known knowledge of the sensors or performing experiments using the same sensor by themselves. However, if the target has properties that are quite different from the knowledge obtained by the attackers, the attacks become more difficult, the evaluation of the attacks in the case that the properties of the target are quite different from the attackers’ knowledge.

In this study, we also assume that the attacker has enough information on the target models. The attacker, however, may have only insufficient information on the target model. Especially, the target model may become different if it is updated. The adversarial examples in the case that the attacker does not have information on the target models have also been discussed [29], and the attacks combining the approach in this paper with such methods are possible. Demonstrating such attacks is one of our future research topics.

Though we need further research to demonstrate the attack in the other cases, the results of this study indicate that the service provider using a machine learning model based on multiple sensors should consider the case in which some of the sensors may be hacked by the attacker. By considering these attacks, we may be able to construct robust models. One of the approaches to constructing robust models is to use the adversarial training, considering the attacks [9]. However, the robustness of adversarial training against such attacks has not yet been discussed, and further research is required. Another approach against an attack from a part of the hacked sensor is to utilize the properties of this attack. Because the attacker cannot access the other sensors, the generated signals may include some inconsistency between the signals from the other sensors. These countermeasures will be a future research topic.

In this study, we investigated the properties of generated attacks. The results indicate that the attacker does not need to generate input signals that are similar to the actual features of the target class. However, these results do not indicate that the signals generated by hacking a small number of sensors are different from the actual features. By training the generator, considering the difference from the actual features of the target class, it may be possible to generate attacks that are difficult to detect based on the difference from the normal features of the target class. Therefore, further research is required to clarify the properties of attacks that cannot be avoided by attackers.

## 6. Conclusions

In this study, we discussed the possibility of attacks on DNN models by hacking a small number of sensors. In this scenario, the attacker first hacks few sensors; then, the attacker can obtain the values of the hacked sensors and change them, but the attacker cannot obtain and change the values of the other sensors.

In this study, we introduced a generator that generates adversarial examples when a small number of sensor devices are hacked. The generator uses the values from the hacked sensors as inputs and generates perturbations so that the features, including the perturbations, are classified into the target class by the target model.

We demonstrated the attack using an open dataset for HAR based on three sensor devices located on the chest, wrist, and ankle of the subjects. We then clarified that the attacker can change the output of the target model by hacking only one of the three devices.

Our future research topics include further research on the properties of attacks, such as countermeasures against attacks.

## Figures and Tables

**Table 1:** Advantages and disadvantages of previous methods.

Researchers	Name of the Proposed Method	Advantages	Disadvantages
Goodfellow et al. [9]	FGSM	One of the first attack methods in the domain of adversarial examples	All features are required; low attack success rate
Kurakin et al. [15]	BIM	Higher attack success ratio than FGSM	All features are required; many iterations are required
Papernot et al. [16]	Saliency map	Attack with a small perturbation	All features are required; computationally expensive
Carlini and Wagner [17]	C&W	Higher attack success ratio than FGSM, BIM, and saliency map	All features are required; computationally expensive
Xiao et al. [18] Jandial et al. [19] Liu et al. [20] Kim et al. [21]	GAN	Higher attack success ratio than FGSM and C&W in the case that adversarial training is used to protect the target model; the perturbations can be generated immediately by using the pre-trained generator.	All features are required; training is required before generating the attack
This paper	Trained generator	The attack can be generated even when the attacker can monitor only a part of the features; the perturbations can be generated immediately by using the pre-trained generator	Training is required before generating the attack

**Table 2:** The advantages and disadvantages of demonstrated adversarial attacks using existing methods.

Researchers	Methods are Used to Generate Attacks	Target Features	Features Required to Be Monitored and Changed	Detail
Finlayson et al. [22]	BIM	Images	All	Demonstration of adversarial examples against medical AI systems
Han et al. [23]	FGSM and BIM	Raw sensor values	All	Demonstration of adversarial examples on raw EEG signals; the generated signals cannot be distinguished from original ECG signals and can fool the target DNN model
Benegui et a. [12]	FGSM and saliency map	Images	All	The first study attempts to quantify the effect of adversarial assaults on machine learning models used for motion sensor-based user identification
Sah et al. [13]	FGSM and BIM	Raw sensor values	All	Demonstration of the transferability of adversarial examples on machine learning models based on wearable sensors
This paper	Trained generator	Raw sensor values	Part	Demonstrates adversarial examples for the case that only a part of the features is monitored by the attacker

**Table 3:** Description of sensors.

Sensor	Locate	Abbreviated
Acceleration from the chest sensor (X axis)	On Chest	Acx
Acceleration from the chest sensor (Y axis)		Acy
Acceleration from the chest sensor (Z axis)		Acz
Electrocardiogram signal (lead 1)		EL1
Electrocardiogram signal (lead 2)		EL2
Acceleration from the left-ankle sensor (X axis)	On Ankle	Alax
Acceleration from the left-ankle sensor (Y axis)		Alay
Acceleration from the left-ankle sensor (Z axis)		Alaz
Gyro from the left-ankle sensor (X axis)		Glax
Gyro from the left-ankle sensor (Y axis)		Glay
Gyro from the left-ankle sensor (Z axis)		Glaz
Magnetometer from the left-ankle sensor (X axis)		Mlax
Magnetometer from the left-ankle sensor (Y axis)		Mlay
Magnetometer from the left-ankle sensor (Z axis)		Mlaz
Acceleration from the right-lower-arm sensor (X axis)	On Wrist	Arlax
Acceleration from the right-lower-arm sensor (Y axis)		Arly
Acceleration from the right-lower-arm sensor (Z axis)		Arlz
Gyro from the right-lower-arm sensor (X axis)		Grlax
Gyro from the right-lower-arm sensor (Y axis)		Grlay
Gyro from the right-lower-arm sensor (Z axis)		Grlaz
Magnetometer from the right-lower-arm sensor (X axis)		Mrlax
Magnetometer from the right-lower-arm sensor (Y axis)		Mrlay
Magnetometer from the right-lower-arm sensor (Z axis)		Mrlaz

**Table 4:** The number of data for each class and subject used in our experiments.

Subject	Class											
	Standing	Sitting	Lying Down	Walking	Climbing Stairs	Waist Bends Forward	Frontal Elevation of Arms	Knees Bending	Cycling	Jogging	Running	Jump Front and Back
1	3072	3072	3072	3072	3072	3072	3072	3379	3072	3072	3072	1075
2	3072	3072	3072	3072	3072	3174	3328	3430	3072	3072	3072	1024
3	3072	3072	3072	3072	3072	3226	3379	3175	3072	3072	3072	1024
4	3072	3072	3072	3072	3072	3328	3277	3123	3072	3072	3072	1024
5	3072	3072	3072	3072	3072	2765	2868	2714	3072	3072	3072	1024
6	3072	3072	3072	3072	3072	2202	2099	2304	3072	3072	3072	1024
7	3072	3072	3072	3072	3072	3072	2765	2816	3072	3072	3072	1024
8	3072	3072	3072	3072	3072	2151	3021	2560	3021	3072	3072	1024
9	3072	3072	3072	3072	3072	2867	2867	2969	3072	3072	3072	1075
10	3072	3072	3072	3072	3072	2458	2765	2867	3072	3072	3072	1024

**Table 5:** Complete results of the three other models with the target model using different training data.

Ground-Truth Class	Precision				Recall			
	Target Model	Wrist	Ankle	Chest	Target Model	Wrist	Ankle	Chest
Standing	100	96	96	41	100	100	88	48
Sitting	100	99	98	54	100	96	97	54
Lying down	100	96	99	100	100	98	99	100
Walking	100	94	99	99	100	53	99	98
Climbing stairs	99	79	98	98	100	97	99	100
Waist bends forward	100	83	96	100	100	94	99	94
Frontal elevation of arms	98	100	89	87	100	99	97	75
Knees bending	100	76	99	100	96	80	97	66
Cycling	96	98	98	76	100	100	98	98
Jogging	98	99	98	87	93	100	93	94
Running	94	97	93	92	97	99	98	89
Jump front and back	95	97	92	100	97	89	94	94

**Table 6:** Results of the MSE on the model estimator using five and three subjects.

Ground-Truth	Five Subjects	Three Subjects
Standing	25.57	28.18
Sitting	19.11	26.31
Lying down	22.08	26.01
Walking	21.75	25.38
Climbing stairs	21.74	25.50
Waist bends forward	28.69	35.91
Frontal elevation of arms	22.30	28.54
Knees bending	22.24	33.81
Cycling	20.06	26.04
Jogging	22.71	35.52
Running	20.00	35.61
Jump front and back	21.76	34.13

## Data Availability

Not applicable.
